# A Practical Guide to Preparation and Applications of Giant Unilamellar Vesicles Formed via Centrifugation of Water-in-Oil Emulsion Droplets

**DOI:** 10.3390/membranes13040440

**Published:** 2023-04-18

**Authors:** Yiting Zhang, Haruto Obuchi, Taro Toyota

**Affiliations:** 1 Department of Basic Science, Graduate School of Arts and Sciences, The University of Tokyo, 3-8-1 Komaba, Meguro, Tokyo 153-8902, Japan; 2 Universal Biology Institute, The University of Tokyo, 3-8-1 Komaba, Meguro, Tokyo 153-8902, Japan

**Keywords:** giant vesicle, centrifugation, water-in-oil emulsion, artificial cell

## Abstract

Giant vesicles (GVs), which are closed lipid bilayer membranes with a diameter of more than 1 μm, have attracted attention not only as model cell membranes but also for the construction of artificial cells. For encapsulating water-soluble materials and/or water-dispersible particles or functionalizing membrane proteins and/or other synthesized amphiphiles, giant unilamellar vesicles (GUVs) have been applied in various fields, such as supramolecular chemistry, soft matter physics, life sciences, and bioengineering. In this review, we focus on a preparation technique for GUVs that encapsulate water-soluble materials and/or water-dispersible particles. It is based on the centrifugation of a water-in-oil emulsion layered on water and does not require special equipment other than a centrifuge, which makes it the first choice for laboratory use. Furthermore, we review recent studies on GUV-based artificial cells prepared using this technique and discuss their future applications.

## 1. Introduction

Giant vesicles (GVs) primarily consist of amphiphiles and water [1]. Amphiphiles form closed sheet-like structures (called bilayer membranes) in water, with their hydrophobic groups facing each other. GVs are larger than 1 μm in size, while vesicles smaller than 1 μm are termed small vesicles (<100 nm) or large vesicles (100 nm to 1 μm). Artificial vesicles composed of phospholipids are called liposomes and those of amphiphilic polymers are called polymersomes. Vesicles can contain water-soluble materials and/or water-dispersible particles in their inner aqueous space, and vesicular membranes can incorporate and functionalize amphiphilic molecules, including membrane proteins. As GVs are similar in size to cells, they can encapsulate micrometer-sized particles and can be observed under an optical microscope. Using optical microscopy, Reeves and Dowben first clarified that GVs are formed from a dry thin film of phospholipids upon water addition [2]. This process (called the film swelling method) produces giant unilamellar vesicles (GUVs) consisting of a single bilayer membrane and giant multilamellar vesicles (GMVs) consisting of multiple bilayer membranes in a nested state. GMVs have a smaller inner aqueous region than that of GUVs with the same diameter, which reduces the total amount of encapsulated water-soluble substances and/or water-dispersible particles. In addition, during the construction of artificial cells [3], which are cell mimicries in terms of the functions of molecular aggregates, the multiple nested structures of GMVs are likely to cause defects while sensing external stimuli and controlling membrane dynamics to release encapsulated materials. Therefore, GUVs have drawn attention to the construction of artificial cells and model cell membranes [4,5,6,7,8,9,10,11].

**Figure 1:**
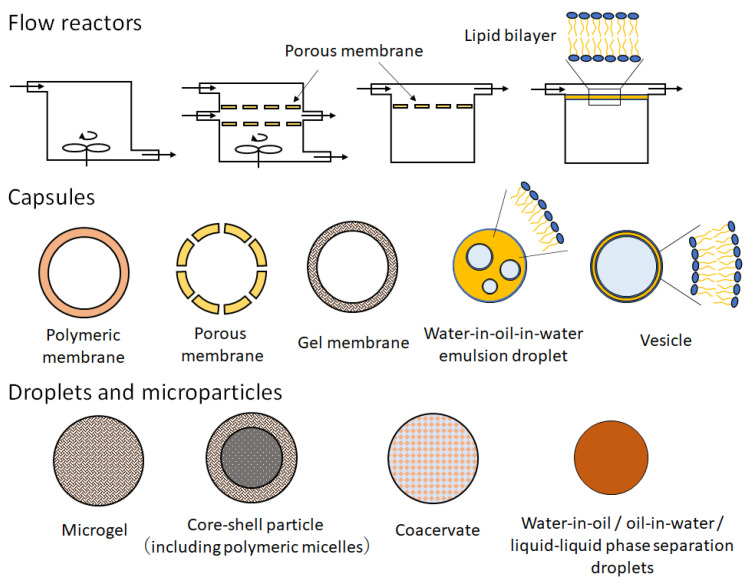
Schematic illustration of micrometer- and submicrometer-sized artificial cells.

Among other artificial cells such as microfabricated flow reactors, micrometer-sized capsules, droplets, and microparticles (Figure 1), GUVs, especially giant unilamellar liposomes, have specific physicochemical properties that resemble those of cells. According to the previous literature and related studies therein [12,13], the membrane thickness and membrane modulus of liposomes are 3–5 nm and 11–30 *k*_B_*T*, respectively. The water permeability in liposomal membrane is 15–150 μm s^−1^. On the other hand, since their constituent molecules are polymer with high intermolecular forces, polymersomes are likely stiffer than liposomes. In fact, their membrane thickness and membrane modulus are 10–50 nm and 40–460 *k*_B_*T*, respectively. The water permeability in polymersomal membranes is 0.7–10 μm s^−1^, which indicates that polymersomal membranes are more hydrophobic than liposomal membranes. When GUVs are composed of biocompatible materials, they can be implanted in vivo. Liposomes as well as polymersomes play an important role in the functionalization of membrane proteins [10]. Therefore, GUV-based artificial cells have recently attracted attention as a new tool for pharmaceutical and medical applications.

**Figure 2:**
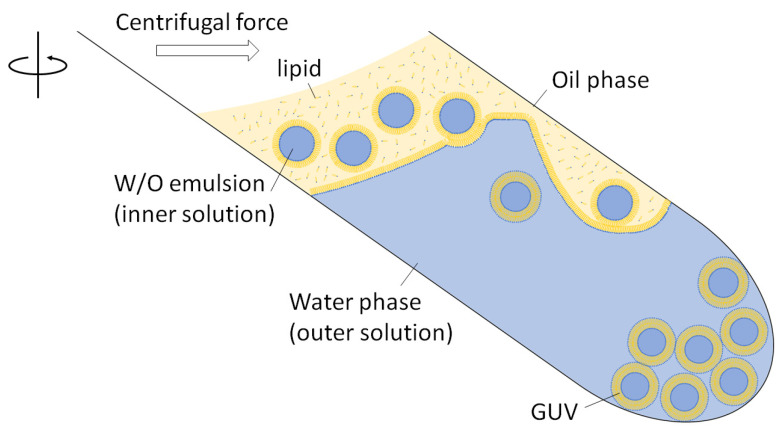
Schematic illustration of GUV preparation via the W/O emulsion template method using centrifugation.

For several decades, preparation methods for GUVs have been investigated. Angelova and Dimitrov applied an alternating electric field during film swelling (called the electroformation method) and observed the formation of GUVs of several dozen micrometers in size [14]. GUVs are possibly formed from a thin film of phospholipids mixed with monosaccharides or polyethylene glycol-tagged phospholipids [15,16]. Using a microcapillary and planar lipid membrane formed by contacting two water drops in oil dissolving phospholipids, uniform GUVs are continuously formed via pulse jetting from the microcapillary (called the pulse jetting method) [17,18]. Another GUV preparation method was developed in which an aqueous solution was suspended in an organic solvent that dissolved amphiphiles, and the organic solvent was gently removed (called the reverse-phase method) [19,20]. In 2003, Pautot et al. proposed that the centrifugation of a water-in-oil (W/O) emulsion (containing phospholipids) layered on an aqueous phase affords GUVs in the aqueous phase [21]. In this method, GUVs are formed via the phase transfer of W/O emulsion droplets when the phospholipid monolayer of the interface comes into contact with another phospholipid monolayer surrounding the droplets under centrifugal force (Figure 2). Although this method does not require any special equipment except a centrifuge, the GUVs formed using this method encapsulate not only water-soluble materials but also water-dispersible micrometer-sized particles at high volume ratios [22].

In recent years, a series of methods have been developed wherein W/O emulsion droplets pass through a lipid monolayer at the oil–water interface to form GUVs. These are called phase transfer methods [23]. To obtain monodispersed GUVs, several studies have established novel hand-made devices and methods, including the continuous droplet interface crossing encapsulation (cDICE) [24] and droplet-shooting and size-filtration (DSSF) methods [25], to control the size of emulsion droplets. Microfluidic devices that generate uniform W/O emulsion droplets and allow them to pass through the water–oil interface have been proposed for GUV preparation [26,27,28]. These methods produce uniform GUV-based artificial cells but require special devices and treatment. Therefore, the W/O emulsion template method proposed by Pautot et al. has the advantage of being simpler and more tunable than other phase transfer methods. This likely makes it the first choice for laboratory use in the construction of GUV-based artificial cells.

## 2. Practical Guide for the Protocol of W/O Emulsion Template Method with Centrifugation

**Figure 3:**
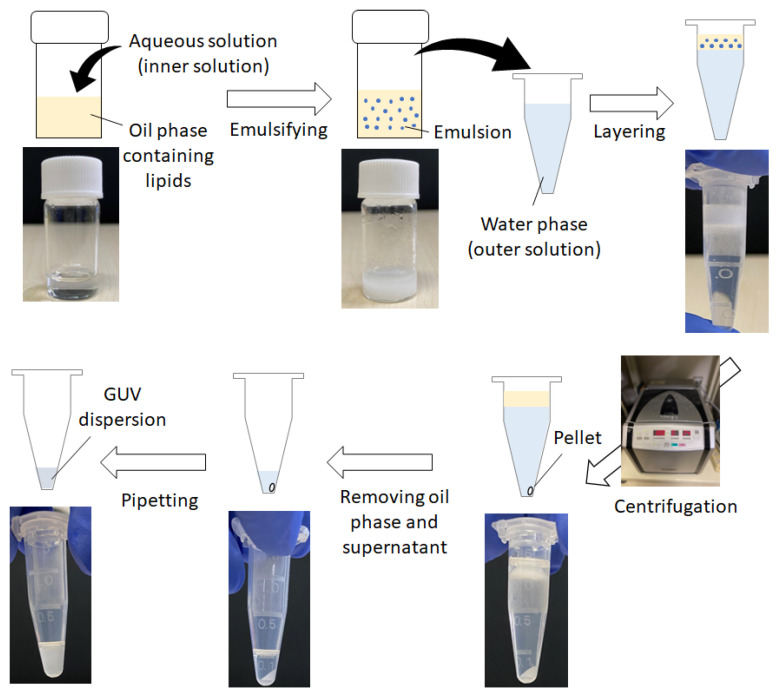
Schematic illustration of the protocol of the W/O emulsion template method with centrifugation.

Figure 3 shows the protocol of the W/O emulsion template method using centrifugation employing a 1.5 mL microtube. The aqueous solution (called the inner solution) suspended in the oil phase should be heavier than the water phase (called the outer solution) below the W/O emulsion, in terms of the principle of centrifugation (an oil phase lighter than the outer solution is adopted). The disproportion between the inner and outer solutions with respect to osmotic pressure and pH is avoided. Thus, the molar concentration of water-soluble materials in both the inner and outer solutions is important. For example, in an experiment employing glucose and sucrose [22], the outer solution dissolved only glucose in a buffered solution, whereas the inner solution was a mixed buffered solution of glucose and sucrose, and the total concentration of sugars in the inner solution was the same as that of glucose dissolved in the outer solution. During the preparation and storage of the oil phase, low humidity is required, and the oil must be kept dry before use. Moga et al. systematically examined GUV yields using various oils and found that mineral oil containing 1-palmitoyl-2-oleoyl-*sn*-glycero-3-phosphocholine (POPC) yielded favorable results for GUV preparation under a certain range of centrifugal forces [29]. Toyota and Zhang also examined the effect of oil (1-octanol, squalene, and liquid paraffin) and its mixture on GUV dispersion and summarized that no significant difference was observed in the case of squalene and liquid paraffin. However, 1-octanol afforded poor yields of GUVs (instead, it produced vesicular aggregates) [30]. It is necessary to explore the optimal concentration and components of lipids dissolved in the oil phase. In several reports on the applications of GUVs [31,32], to reinforce membrane stability and avoid aggregation of vesicles, additive lipids such as cholesterol and polyethylene glycol-tagged phospholipids were mixed at an amount less than that of phosphatidylcholine in the oil. Table 1 is a summary of the materials and experimental conditions in the protocol of the W/O emulsion template method with centrifugation.

The inner solution is first added to the oil phase which contains dissolved lipids. The volume ratio of the inner solution to the oil phase is less than 30%. The lower the volume of the inner solution, the smaller the size of the obtained GUVs [29]. The mixture is emulsified via mechanical agitation such as vortex mixing, hand tapping, rubbing on a rumble strip (e.g., a tube stand), filtration, gentle stirring, and shear mixing with a propeller. The size of the W/O emulsion droplets also depends on the intensity of the mechanical agitation stimulus and the procedures followed by the individual experimenter [29,33]. Ultrasonication is also effective in emulsifying an oil phase with the inner solution [34]. However, the radical species generated upon ultrasonication exposure can chemically damage the encapsulated water-soluble materials and lipid molecules [35]. The W/O emulsion formed via ultrasonication exposure affords smaller droplets, producing GUVs with only several micrometer diameters after centrifugation [21,36].

The obtained W/O emulsion is then gently layered onto the outer solution. The container for this biphase system, such as a test tube, microtube, or 96-well microtiter plate, is selected based on the available volume of the inner solution, especially when dealing with proteins and DNA/RNA for encapsulation. To the best of our knowledge, the container employed for the minimum volume of the inner solution (several microliters) is a 200 µL PCR microtube (a jig is needed to secure the PCR microtubes to the rotor of the high-performance centrifuge) [37]. The biphase system is incubated at a low temperature (e.g., 4 °C) before centrifugation in many cases. Pautot et al. reported that the adsorption rate of lipids on the surface of the W/O emulsion droplets and oil–water interface of biphasic systems is low to reach equilibrium, requiring several hours [21]. However, Moga et al. revealed that the yield and size distribution of the obtained GUVs are not significantly influenced by the incubation time (5–60 min) [29]. Therefore, the incubation process is minimal.

Subsequently, the W/O emulsion droplets are settled and passed through the interface of the biphasic system under centrifugation, resulting in the formation of GUVs. Centrifuge angle rotors have been used for test tubes and microtubes, while swing rotors have also been adopted, especially for microtiter plates. The centrifugation force and time are estimated using the Stokes theorem [22]. The yield of GUVs increased as the centrifugation force (50–1200× *g*) and time (0.5–10 min) increased [29]. This indicates that some GUVs are formed from a deformed water–oil interface, which wets the inner wall of the container during centrifugation (Figure 1). In contrast, the temperature during centrifugation (4–37 °C) does not significantly affect the GUV yield in such a short time. This means that the current method is tunable and can be optimized for the preferred conditions of the encapsulated and/or membrane-anchoring materials.

Finally, GUVs are collected from the pellets that precipitate after centrifugation. Two collection methods have been reported. One method involves opening a tiny hole at the bottom of the tube using a pin or needle, and the drops that fall through the hole are collected in a separate tube [22,30,31,32,38]. The other is aspiration using a micropipette to remove both the oil phase and supernatant of the outer solution, followed by gentle pipetting [30,33,36,37,39,40]. GUVs are gently dispersed using a micropipette to complete the preparation. Optical microscopy is the most commonly used method for GUV observation and evaluation. Phase contrast, differential interference contrast, or dark-field microscopy are adopted to observe non-labeled GUVs, whereas GUVs labeled with encapsulated fluorescent molecules or fluorophore-tagged amphiphiles can be observed under an epifluorescence microscope or a confocal laser scanning fluorescence microscope [41]. Note that in the former observation method for non-labeled GUVs, because the inner solution has a higher refractive index than the lipid bilayer membrane and outer solution, the vesicular membrane itself is often difficult to identify. Hence, the latter methods are used; however, the spatial resolution is too high to track floating GUVs in a short time-lapse. The avidin–biotin interaction is sometimes used for having the GUVs adhere to a substrate [42], and other techniques that modify the surface of the substrate of the specimen [43,44] or bury GUVs in agarose gel [45,46] are required for precise observation.

To construct stable GUV-based artificial cells, Mabrouk et al. reported that polymersomes can be prepared by the W/O emulsion template method via centrifugation [47,48]. Polyethyleneoxide-*b*-polybutadiene (PEO-*b*-PBD) and other amphiphilic block copolymers were independently dissolved into toluene and the inner solution containing a mixture of sucrose and dextran (MW = 41,000) was then emulsified in the toluene solution via gentle pipetting in a short time. The emulsion was poured onto the outer solution of glucose and centrifuged at 100× *g* for 12 min. Marguet et al. also used PEO-*b*-PBD in polymersomes containing hydrophilic polymers [49], liposomes [50], or small polymersomes [51] to mimic cytoplasmic conditions within polymersomes.

The important practical aspects of the evaluation of GUV dispersions are their size and the number of lipid bilayer membranes, called lamellarity, their inner structure containing lipidic particles, and the trace amount of oil contained in the GUV membrane. These are influenced by the aforementioned materials (lipids and oils) and conditions.

**Table 1:** Studies on the materials and experimental conditions used in the W/O emulsion template method protocol with centrifugation.

Ref.	Oil	Inner and Outer Solutions ^1^	Lipids ^2^	Emulsifying Techniques	Container Volume	Centrifugation Conditions	Collecting GUVs
[21]	Dodecane and squalene	NaCl and Tris-HCl	Egg-PC, POPC, POPS, and DOPS	Filtration, gentle stirring, shear mixing, and ultrasonication	50 mL	5–10 min, unknown temp., and 120× *g*	–
[22]	Liquid paraffin	Glucose + sucrose, glucose, and Tris-HCl	DOPC	Shear mixing	1.5 mL	30 min, 20 °C, and 18,800× *g*	Opening hole
[29]	Mineral oil, 1-octanol, oleic acid, anisole, silicon oil, and squalene	Sucrose, glucose, Tris-HCl, HEPES-NaOH, and PBS	POPC	Rubbing on a rumble strip, vortex mixing, hand tapping, and ultrasonication	300 μL ^3^	0.5–10 min, 4–37 °C, and 50–1200× *g*	N/A ^4^
[30]	1-Octanol, squalene, and liquid paraffin	Sucrose, glucose, and HEPES-NaOH	POPC + POPG	Rubbing on a rumble strip	1.5 mL	30 min, 20 °C, and 18,800× *g*	Opening hole or pipette aspiration
[31]	Liquid paraffin	Sucrose and NaCl + Tris-HCl	Egg-PC + DMPG + cholesterol	Hand tapping	1.5 mL	30 min, 20 °C, and 18,800× *g*	Opening hole
[32]	Liquid paraffin + squalene	Sucrose, glucose, and NaCl + Tris-HCl	Egg-PC + PEG5000-DOPE + cholesterol	Hand tapping	1.5 mL	30 min, 0 °C, and 18,800× *g*	Opening hole
[33]	Mineral oil	Sucrose, glucose, and HEPES-NaOH	POPC	Rubbing on a rumble strip, vortex mixing, and ultrasonication	1.5 mL	15 min, r.t. ^5^, and 16,100× *g*	Pipette aspiration
[36]	Mineral oil	Sucrose and glucose	POPC + PEG2000-DSPE	Ultrasonication and gentle stirring	300 μL ^3^	–	Pipette aspiration
[37]	Liquid paraffin + squalene	PIPES-HCl + MgCl_2_ + EGTA	DOPC + PEG2000-DSPE + cholesterol	Hand tapping	200 μL	30 min, 4 °C, and 18,800× *g*	Pipette aspiration
[38]	Liquid paraffin	Sucrose and glucose	POPC + cholesterol	Vortex mixing	1.5 mL	30 min, 4 °C, and 9000× *g*	Opening hole
[39]	Squalene	Sucrose, glucose, and Tris-HCl	Polyglycerol- polyricinoleate	Hand tapping	15 mL	30 min, r.t. ^5^, and 2250× *g*	Pipette aspiration
[40]	Mineral oil	Sucrose, glucose, and HEPES-NaOH	POPC + cholesterol	Vortex mixing	15 mL	10 min, 4 °C, and 100× *g*/400× *g* ^6^	Pipette aspiration
[47]	Toluene	Sucrose + dextran and glucose	PEO-*b*-PBD, PEG-*b*-PA444, and PEG-*b*-PA6ester1	Gentle pipetting	1.5 mL ^7^	12 min, unknown temp., and 100× *g*	–
[49]	Toluene	Sucrose and glucose	PEO-*b*-PBD	Hand tapping	1.5 mL ^7^	5 min, r.t. ^5^, and 500× *g*	–

^1^ For inner solution, only major water-soluble components are listed in the table. Abbreviations: Tris-HCl; tris(hydroxymethyl)aminomethane buffered solution with hydrogen chloride, HEPES-NaOH; 2-[4-(2-hydroxyethyl)-1-piperazinyl]ethanesulfonic acid buffered solution with sodium hydroxide, PIPES-HCl; piperazine-*N*,*N*′-bis(2-ethanesulfonic acid) buffered solution with hydrogen chloride, PBS; phosphate-buffered saline, and EGTA; ethylenebis(oxyethylenenitrilo)tetraacetic acid. ^2^ Abbreviations: egg-PC; egg-phosphatidylcholine, POPC; 1-palmitoyl-2-oleoyl-*sn*-glycero-3-phosphocholine, DOPC; 1,2-dioleoyl-*sn*-glycero-3-phosphocholine, POPG; 1-palmitoyl-2-oleoyl-*sn*-glycero-3-phosphoglycerol sodium salt, DMPG; 1,2-dimyristoyl-*sn*-glycero-3-phosphoglycerol sodium salt, POPS; 1-palmitoyl-2-oleoyl-*sn*-glycero-3-phospho-_L_-serine, DOPS; 1,2-dioleoyl-*sn*-glycero-3-phospho-_L_-serine, PEG2000-DSPE; 1,2-distearoyl-*sn*-glycero-3-phosphoethanolamine-*N*-[methoxy(polyethyleneglycol)-2000], PEG5000-DOPE; 1,2-dioleoyl-*sn*-glycero-3-phosphoethanolamine-*N*-[methoxy(polyethyleneglycol)-5000], PEO-*b*-PBD; Polyethyleneoxide-*b*-polybutadiene block copolymer, MW 7300 (DP_n_, 52 (PEO), 93 (PBD)), M_w_/M_n_ 1.04, 89% 1,2-addition of butadiene, PEG-*b*-PA444; polyethylene glycol-*b*-poly (4′-acryloxybutyl-2,5-di-(4′-butyloxynbenzoylox)benzoate block copolymer, MW 6700 (DP_n_, 45 (PEG), 7 (PA444)), M_w_/M_n_ 1.09, and PEG-*b*-PA6ester1; polyethylene glycol-*b*-poly(4-methoxyphenyl-4-(6-acryloyloxyhexyloxy)benzoate block copolymer, MW 7900 (DP_n_, 45 (PEG), 15 (PA6ester1)), M_w_/M_n_ 1.13. ^3^ A 96-well microtiter plate was used. ^4^ The 96-well microtiter plate equipped with a thin glass bottom was used for direct observation via an inverted optical microscope without collecting GUVs. ^5^ Room temperature. ^6^ The samples were centrifuged for 10 min at 100× *g* and 4 °C and then again with an altered force (400× *g*) for 10 min at 4 °C. ^7^ There is no explicit explanation on the container volume in the references. However, the volumes of emulsion and the outer solution in the tube were 50–60 μL and 30 μL, respectively, and the researchers used an Eppendorf centrifuge (5417R) with an angle rotor for 1.5/2.0 mL tubes. Therefore, we indicate that the container volume was 1.5 mL.

**Figure 4:**
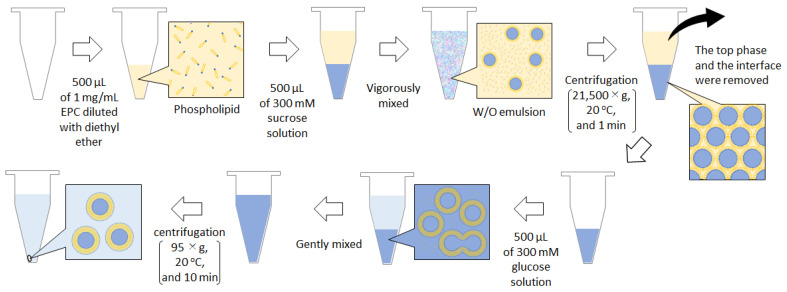
Schematic illustration of the protocols of the W/O emulsion template method focusing on the effective removal of oil.

Table 1 shows the inner and outer solutions, emulsifying techniques, and centrifugation conditions. The lamellarity of the obtained GUVs is determined using some observational methods. The relative membrane multiplicity can be evaluated from the fluorescence intensity of each vesicle in the dispersion [52]. However, fluorescence microscopy is not sufficient to evaluate a single bilayer membrane; thus, Yamazaki proposed a combination of fluorescence microscopy observations with glass capillary aspiration to measure the membrane bending modulus [53]. Any leakage of the inner solution, which contains a dye, can be determined via exposure to α-hemolysin, a membrane pore-forming protein [54]. As α-hemolysin cannot permeate into the inner solution but is incorporated into the outmost vesicular membrane, the leakage of the inner solution indicates that the leaked vesicles are attributable to GUVs. The membranes of GUVs usually contain trace amounts of oil in the intramembrane region, as indicated by water permeation measurements [55] and micro-Raman spectroscopy [56]. Moreover, lipidic particles (usually smaller than 1 μm) can be observed both inside and outside the GUVs. Pautot et al. reported that the droplets in the W/O emulsion contain lipidic particles (small or large vesicles) and that, as a result, GUVs containing these particles are formed [21]. In addition, in the GUV pellet under centrifugal force, the vesicular membranes of some GUVs can collapse and change into lipidic particles as a ternary mixture of lipid/water/oil [57]. If these particles are formed and GUVs are scarcely observed in a specimen prepared using a previously reported protocol, attention should be paid to the degradation of oil and/or lipids, and the emulsification and centrifugation conditions should be readjusted accordingly [58]. An alternative W/O emulsion template method with centrifugation, which suppresses the formation of such lipidic particles, is also useful for the preparation of GUVs [59]. This method is proposed as a more efficient method combining centrifugation with the reverse-phase method for GUV preparation, which focuses on the effective removal of oil from a biphasic system (Figure 4). Diethyl ether is not only volatile but also a good solvent for lipids and is lighter than water. By centrifuging the suspension of lipid-dissolved diethyl ether layered on the outer solution, while the emulsion droplets settle, the water separates to the lower layer of the tube and diethyl ether to the upper layer. As a result, an aggregate of W/O emulsion droplets neighboring each other and forming a bilayer membrane is precipitated by the movement of such liquids. GUVs are formed by mixing and stirring the solution to the packed precipitates of the droplets produced during the transfer of W/O emulsion droplets. Therefore, this technique, combining the two preparation methods for GUVs, provides a good GUV yield and leaves a low amount of organic solvent residue in the GUV intramembrane.

## 3. Construction and Application of GUV-Based Artificial Cells

According to the pioneering research by Chang et al. [3], artificial cells are conceived as microscopic particles with semipermeable boundaries that are separated from the surrounding aqueous environment. In particular, capsule-type artificial cells have the advantage of being able to exchange energy and water-soluble substances between external and internal regions (Figure 1). Polymeric and/or porous membranes have been used in artificial cells owing to their chemical and physical stability. In contrast, GUV-based artificial cells have drawn increasing attention because the vesicular membrane plays the role of a scaffold for membrane proteins and because GUVs can encapsulate micrometer-sized particles such as organelles and even cells [60,61,62,63,64]. Furthermore, by combining giant vesicles with different functions, the construction of artificial tissues that mimic multicellular systems with chemical communications among the artificial cells has been reported [65,66,67,68,69]. The W/O emulsion template method with centrifugation contributed to the preparation of the functionalized GUVs.

### 3.1. Repetitive Transformation via Protein Polymerization and Depolymerization

Hayashi et al. reported that the deformation of GUVs based on the polymerization of the encapsulated tubulin could be controlled via the degree of hydrostatic pressure [37]. The GUVs were monitored using a high-pressure apparatus on the microscope stage. At atmospheric pressure (0.1 MPa), many GUVs exhibited protrusions because of tubulin polymerization, whereas the application of high pressure (60 MPa) caused protrusions to contract via tubulin depolymerization in tens of seconds. This process was repeated three times, and the protrusions were regenerated within minutes after the pressure was released. This repetitive behavior indicated that the motility of the GUVs is potentially driven by internal protein polymerization. Takiguchi et al. examined GUVs containing F-actin and heavy meromyosin (HMM) and observed that the deformation of GUVs depended on the formation of the F-actin cross-linking network from HMM [70]. Moreover, they found that GUVs could be stretched or retracted by turning the excitation light for the fluorescent molecules labeled on F-actin on and off [71]. As light irradiation causes the fluorescent molecules to generate radicals that disrupt the polymerization of F-actin, the membrane tension of the GUVs stretched by F-actin is released and the GUVs return to their spherical shape. F-actin then returns to its original elongated state via repolymerization without light irradiation, and the GUVs are elongated again. As the radicals act on F-actin, up to eight repetitions of the elongation and contraction of the GUVs were observed in their experiments. These deformable GUVs can be applied to soft actuators.

### 3.2. Bioreactor for Cell-Free Protein Synthesis

**Figure 5:**
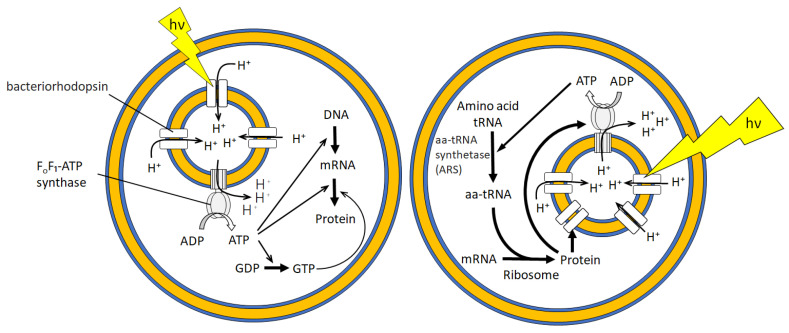
Schematic illustration of artificial photosynthetic cells exhibiting protein (GFP) synthesis inside GUV (**left**) and self-constituting protein synthesis (**right**) driven by light.

Green fluorescent protein (GFP) is a typical indicator of the completion of transcription–translation reaction employing GFP-coded DNA or RNA. Nishimura et al. reported the analysis of the extent of GFP expression by encapsulating a cell-free protein translation reagent and GFP-coded RNA inside GUVs [72]. They assessed the influence of GUV size, GUV membrane components, and the presence or absence of GUVs in a cell-free protein synthesis solution. They did not find any differences in the effects of these factors but observed that the internal and external environments of the GUVs affected the expression of GFP. Noireaux et al. created a bioreactor in which α-hemolysin was co-expressed with GFP in GUVs [73]. Once cell-free protein synthesis reagents were depleted inside the GUVs, GFP was no longer synthesized. However, this bioreactor allowed the reagents to be supplied from the outside because α-hemolysin, a membrane pore, was formed in the vesicular membrane. Therefore, GFP expression in this bioreactor was maintained for 4 days. Recently, the encapsulation of liposomes bearing F_o_F_1_-ATP synthase and bacteriorhodopsin inside GUVs was achieved [74]. These GUV-based artificial cells perform the photosynthesis of ATP and protein (GFP) synthesis upon light irradiation (Figure 5). These studies demonstrated that the compartmentalization and internalization of such reagents can be achieved using a 5 nm thick bilayer membrane that is non-covalently formed.

### 3.3. DNA Nanotechnology Platform

Nanotechnology using DNA has rapidly developed for several decades in molecular computing and molecular precise architecture [69]. When such DNA systems are incorporated into GUVs, a synergic effect of the vesicular membrane and DNA is expected. For example, Sato et al. demonstrated that, because of the compartmentalization of GUVs, a DNA amplification circuit therein can provide a keen signal amplifier sensing a tiny amount of input DNA [75]. They also reported an amoeba-like actuator based on a GUV governed by DNA hybridization [76]. As the GUV sensed UV light as an input and exhibited the amoeba-like deformation as an output, it was called an amoeba-type molecular robot. The components of this molecular robot are DNA that can form new duplexes in response to UV light irradiation, kinesin, a motor protein to which DNA with a complementary base sequence of the UV-responsive DNA is bound, microtubule, a fibrous protein to which kinesin binds and moves, and GUV containing DNA-tagged cholesterol. UV light irradiation caused DNA to bind kinesin to the vesicular membrane, and the resulting sliding motion of kinesin on the microtubules is transmitted to the membrane (i.e., the DNA acts as a “clutch”), resulting in deformation of the GUV.

### 3.4. Fusion and Fission Assisted by a Microfabricated Platform

Shiomi et al. proposed a micro-dose biochemical-reagent-dispensing platform that enabled two GUVs to merge via electrofusion and then re-disperse [77]. One GUV contained enzymes and fluorescent substances, while the other contained calcein/cobalt and ethylenediaminetetraacetic acid (EDTA). The fusion of the two GUVs showed controllability when biochemical reagents were mixed via fluorescence emission and quenching based on enzyme reactions. Terasawa et al. reported that GUVs newly formed by the electrofusion of GUVs are thermodynamically unstable and simultaneously exhibit fission while constraining polyethylene glycol [78]. This characteristic transformation is governed by an entropic effect, called the depletion force. Schmid et al. demonstrated the chemically driven membrane fusion of GUVs containing negatively charged phospholipids with cell membranes upon calcium ion addition [79]. Electrofusion of GUVs and cells was also reported for transporting materials into cells [80].

### 3.5. Encapsulation of Micrometer-Sized Particles

As a GUV provides a closed space, the encapsulated polymer and particles exhibit entropically driven phenomena. Natsume et al. reported that when micrometer-sized polystyrene particles of two sizes are encapsulated in a GUV, size-dependent localization of the particle distribution occurs [81]. The GUV contained several hundred large particles, whose volume fraction exceeded that of the small particles. Although it was estimated that the depletion force exerted by the small particles was approximately identical to that exerted by the large particles, the small particles were found to be near the vesicular membrane. They also observed the deformation of GUVs encapsulating charged particles by optimizing the arrangement relationship of the charged particles inside the GUV [82]. Further, they found that the GUV is a model for interpreting cell deformation caused by the crowding effect related to the increase in free volume under geometric conditions.

The W/O emulsion template method with centrifugation additionally enables the encapsulation of functionalized particles inside GUVs. Suzuki et al. constructed GUVs that encapsulated smaller giant vesicles containing synthesized caged phospholipids into their membrane [83]. Immediately after the exposure of the nested GUV to UV irradiation, the smaller giant vesicles released their inclusion, dsDNA, and strong fluorescence was observed in the inner solution of the GUV, indicating that the dsDNA had complexed with a fluorescent probe. This release technique, which employs caged phospholipids, can be used to obtain a photo-responsive initiator for cell-free protein synthesis inside GUVs.

### 3.6. Intercellular Communication Model

Even the challenges of constructing GUV-based artificial organisms have begun for mimicking cell differentiation and hierarchical organization. The W/O emulsion template method by centrifugation contributes to the preparation of GUVs that can communicate with each other by diffusing chemical signals. So far, the researchers made these chemical signals pass through α-hemolysin, a pore-forming membrane protein, that is incorporated into the vesicular membrane. For example, Bolognesi et al. constructed donor and acceptor GUVs bearing α-hemolysin [84]. The donor and acceptor contained calcium ions and calcium-sensitive fluorescent dye, Fluo-4, respectively. In the outer solution, the cyclodextrin blocker (heptakis(2,3,6-tri-O-methyl)-β-cyclodextrin) was added to prevent leakage through α-hemolysin. Since the blocker is slightly larger than the intermembrane distance between GUVs neighbored by an optical tweezer, its ability to penetrate this space was diminished, allowing selective translocation of calcium ions from the donor to the acceptor. Buddingh’ et al. realized a population intercellular communication using sender and receiver GUVs containing α-hemolysin [85]. The sender GUVs encapsulated enzymatic reaction reagents for AMP synthesis from ATP, and the receiver incorporated enzymatic reaction circuits for NADH synthesis allosterically activated by AMP. This intercellular communication in the GUV population of a mixture of the senders and receivers successfully performed diffusible signaling of AMP over a long distance of 5 mm.

### 3.7. Stimulus-Responsive Sensor and Releaser

In addition to the small and/or large vesicles used in drug delivery systems, GUVs have been paid attention in medical applications. Hayashi et al. attempted to produce a tissue marker for location identification during surgical navigation [39,86,87]. They encapsulated large vesicles bearing an infrared fluorescent dye in a polymeric GUV marker developed to locate gastric cancer. Moreover, they proposed a system for drug release upon ultrasonic irradiation, a device used in clinical practice, and investigated its release conditions [39].

To evaluate biological clues, Su et al. developed a sensing mechanism in which the fluorescence from inside the GUV was enhanced by the attachment of antibody-like proteins to the vesicular membrane [88]. The GUVs contained cell-free protein synthesis reagents that produce proteins that can trigger antigen–antibody reactions corresponding to specific proteins. The GUVs selectively fluoresced in the presence of binding proteins, making them useful for the selective detection of biomolecules.

Hamada et al. synthesized an insect pheromone receptor, a complex of membrane proteins BmOR1 and BmOrco, by internalizing mRNA and cell-free protein synthesis reagents into GUVs [32]. When insect pheromones were added to the GUVs, significant ion current activity was observed using the whole-cell patch clamp technique, indicating that the pheromone receptor was incorporated into the vesicular membrane. Xu et al. produced a 16-helix transmembrane channel pore, which was completely de novo designed, on GUV membranes [89]. The GUVs passed biotin tagged with fluorescent dyes through the channel pores, and cryo-transmission electron microscopy revealed that the structure of the channel pores was consistent with that of the designed model.

**Figure 6:**
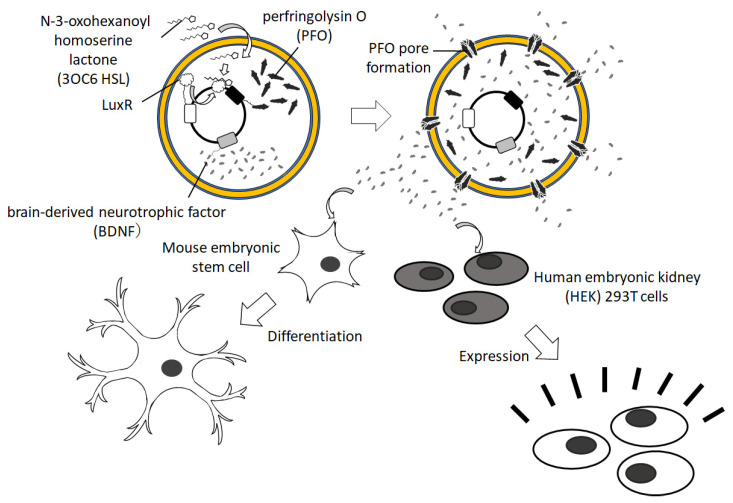
Schematic illustration of artificial cells driving neural differentiation and cell transformation.

For the medical application of GUVs encapsulating cell-free protein synthesis reagents, a cancer drug was conceived by Krinsky et al. [90]. They developed GUVs capable of synthesizing Pseudomonas exotoxin A, which exhibited antitumor activity. When GUVs were administered to a mouse model of 4T1 tumors, the histological evaluation revealed strong apoptosis in the tumor tissue. Toparlak et al. proposed a new GUV-based artificial cell that could send chemical signals (Figure 6) [91]. They encapsulated cell-free protein synthesis reagents and a plasmid encoding membrane pore and mediator proteins for signaling to other cells in the GUVs. These GUVs could promote neuronal differentiation and growth and induce GFP synthesis in various cell types.

## 4. Summary

In this review, we brought up GUV preparation methods employing W/O emulsion and centrifugation. These methods do not require any special equipment other than a benchtop centrifuge, which is commonly used in materials and life sciences laboratories. Using the prepared GUVs as artificial cells, we can analyze physicochemical and biochemical phenomena related to the cellular system. This method has potential in pharmaceutical applications where GUVs sense chemical and physical stimuli and release drugs.

## Data Availability

Not applicable.

## References

[1] Dimova R., Stano P., Marques C.M., Walde P.B., Dimova R., Marques C.M. (2020). Preparation methods for giant unilamellar vesicles. The Giant Vesicle Book.

[2] Reeves J.P., Dowben R.M. (1969). Formation and properties of thin-walled phospholipid vesicles. J. Cell Physiol..

[3] Chang T.M.S. (1995). Artificial cells with emphasis on bioencapsulation in biotechnology. Biotech. Ann. Rev..

[4] Jiang W., Wu Z., Gao Z., Wan M., Zhou M., Mao C., Shen J. (2022). Artificial Cells: Past, Present and Future. ACS Nano.

[5] Lu Y., Cho E. (2020). Compartmentalizing Cell-Free Systems: Toward Creating Life-Like Artificial Cells and Beyond. ACS Synth. Biol..

[6] Jeong S., Nguyen H.T., Kim C.H., Ly M.N., Shin K. (2020). Toward Artificial Cells: Novel Advances in Energy Conversion and Cellular Motility. Adv. Funct. Mater..

[7] Matosevic S. (2012). Synthesizing artificial cells from giant unilamellar vesicles: State-of-the art in the development of microfluidic technology. BioEssays.

[8] Buddingh’ B.C., van Hest J.C.M. (2017). Artificial Cells: Synthetic Compartments with Life-like Functionality and Adaptivity. Acc. Chem. Res..

[9] Ai Y., Xie R., Xiong J., Liang Q. (2020). Microfluidics for Biosynthesizing: From Droplets and Vesicles to Artificial Cells. Small.

[10] Lo C.H., Zeng J. (2023). Application of polymersomes in membrane protein study and drug discovery: Progress, strategies, and perspectives. Bioeng. Transl. Med..

[11] Messager L., Gaitzsch J., Chierico L., Battaglia G. (2014). Novel aspects of encapsulation and delivery using polymersomes. Curr. Opin. Pharmacol..

[12] Le Meins J.-F., Sandre O., Lecommandoux S. (2011). Recent trends in the tuning of polymersomes’ membrane properties. Eur. Phys. J. E.

[13] Méléard P., Gerbeaud C., Pott T., Fernandez-Puente L., Bivas I., Mitov M.D., Dufourcq J., Bothorel P. (1997). Bending elasticities of model membranes: Influences of temperature and sterol content. Biophys. J..

[14] Angelova M.I., Dimitrov D.S. (1986). Liposome electroformation. Faraday Discuss. Chem. Soc..

[15] Tsumoto K., Matsuo H., Tomita M., Yoshimura T. (2009). Efficient formation of giant liposomes through the gentle hydration of phosphatidylcholine films doped with sugar. Colloids Surf. B Biointerfaces.

[16] Yamashita Y., Oka M., Tanaka T., Yamazaki M. (2002). A new method for the preparation of giant liposomes in high salt concentrations and growth of protein microcrystals in them. Biochim. Biophys. Acta-Biomembr..

[17] Funakoshi K., Suzuki H., Takeuchi S. (2007). Formation of giant lipid vesiclelike compartments from a planar lipid membrane by a pulsed jet flow. J. Am. Chem. Soc..

[18] Stachowiak J.C., Richmond D.L., Li T.H., Liu A.P., Parekh S.H., Fletcher D.A. (2008). Unilamellar vesicle formation and encapsulation by microfluidic jetting. Proc. Natl. Acad. Sci. USA.

[19] Walde P., Cosentino K., Engel H., Stano P. (2010). Giant vesicles: Preparations and applications. ChemBioChem.

[20] Moscho A., Orwar O.W.E., Chiu D.T., Modi B.P., Zare R.N. (1996). Rapid preparation of giant unilamellar vesicles. Proc. Natl. Acad. Sci. USA.

[21] Pautot S., Frisken B.J., Weitz D.A. (2003). Production of Unilamellar Vesicles Using an Inverted Emulsion. Langmuir.

[22] Natsume Y., Toyota T. (2013). Giant vesicles containing microspheres with high volume fraction prepared by water-in-oil emulsion centrifugation. Chem. Lett..

[23] Faizi H.A., Tsui A., Dimova R., Vlahovska P.M. (2022). Bending rigidity, capacitance, and shear viscosity of giant vesicle membranes prepared by spontaneous swelling, electroformation, gel-assisted, and phase transfer methods: A comparative study. Langmuir.

[24] Abkarian M., Loiseau E., Massiera G. (2011). Continuous droplet interface crossing encapsulation (cDICE) for high throughput monodisperse vesicle design. Soft Matter.

[25] Morita M., Onoe H., Yanagisawa M., Ito H., Ichikawa M., Fujiwara K., Saito H., Takinoue M. (2015). Droplet-Shooting and Size-Filtration (DSSF) Method for Synthesis of Cell-Sized Liposomes with Controlled Lipid Compositions. ChemBioChem.

[26] Siddharth D., Dekker C. (2018). On-chip microfluidic production of cell-sized liposomes. Nat. Protoc..

[27] Yandrapalli N., Petit J., Bäumchen O., Robinson R. (2021). Surfactant-free production of biomimetic giant unilamellar vesicles using PDMS-based microfluidics. Commun. Chem..

[28] Ushiyama R., Koiwai K., Suzuki H. (2022). Plug-and-play microfluidic production of monodisperse giant unilamellar vesicles using droplet transfer across Water-Oil interface. Sens. Actuators B Chem..

[29] Moga A., Yandrapalli N., Dimova R., Robinson T. (2019). Optimization of the inverted emulsion method for high-yield production of biomimetic giant unilamellar vesicles. ChemBioChem.

[30] Toyota T., Zhang Y. (2022). Effect of an Oil Medium on Giant Vesicles Prepared with Water-in-Oil Emulsion. Bunseki Kagaku.

[31] Toyota T., Ohguri N., Maruyama K., Fujinami M., Saga T., Aoki I. (2012). Giant vesicles containing superparamagnetic iron oxide as biodegradable cell-tracking MRI probes. Anal. Chem..

[32] Hamada S., Tabuchi M., Toyota T., Sakurai T., Hosoi T., Nomoto T., Nakatani K., Fujinami M., Kanzaki R. (2014). Giant vesicles functionally expressing membrane receptors for an insect pheromone. Chem. Commun..

[33] Matsushita-Ishiodori Y., Hanczyc M.M., Wang A., Szostak J.W., Yomo T. (2019). Using imaging flow cytometry to quantify and optimize giant vesicle production by water-in-oil emulsion transfer methods. Langmuir.

[34] Ashokkumar M. (2011). The characterization of acoustic cavitation bubbles—An overview. Ultrason Sonochem..

[35] Yin H., Xu L., Porter N.A. (2011). Free radical lipid peroxidation: Mechanisms and analysis. Chem. Rev..

[36] Hadorn M., Eggenberger P. Towards Personalized Drug Delivery-Preparation of an Encapsulated Multicompartment System. Towards Personalized Drug Delivery. Proceedings of the Third International Conference on Biomedical Electronics and Devices.

[37] Hayashi M., Nishiyama M., Kazayama Y., Toyota T., Harada Y., Takiguchi K. (2016). Reversible morphological control of tubulin-encapsulating giant liposomes by hydrostatic pressure. Langmuir.

[38] Fujii S., Matsuura T., Sunami T., Nishikawa T., Kazuta Y., Yomo T. (2014). Liposome display for in vitro selection and evolution of membrane proteins. Nat. Protoc..

[39] Yahagi R., Yoshida K., Zhang Y., Ebata M., Toyota T., Yamaguchi T., Hayashi H. (2016). Destruction of giant cluster-like vesicles by an ultrasonically activated device. Jpn. J. Appl. Phys..

[40] Adir O., Sharf-Pauker N., Chen G., Kaduri M., Krinsky N., Shainsky-Roitman J., Shklover J., Schroeder A. (2020). Preparing Protein Producing Synthetic Cells using Cell Free Bacterial Extracts, Liposomes and Emulsion Transfer. J. Vis. Exp..

[41] Toyota T., Zhang Y. (2022). Identifying and Manipulating Giant Vesicles: Review of Recent Approaches. Micromachines.

[42] Sackmann E. (1996). Supported membranes: Scientific and practical applications. Science.

[43] Gruhn T., Franke T., Dimova R., Lipowsky R. (2007). Novel method for measuring the adhesion energy of vesicles. Langmuir.

[44] Rädler J.O., Feder T.J., Strey H.H., Sackmann E. (1995). Fluctuation analysis of tension-controlled undulation forces between giant vesicles and solid substrates. Phys. Rev. E.

[45] Lira R.B., Steinkühler J., Knorr R.L., Dimova R., Riske K.A. (2016). Posing for a picture: Vesicle immobilization in agarose gel. Sci. Rep..

[46] Tsumoto K., Oohashi M., Tomita M. (2011). Monitoring of membrane collapse and enzymatic reaction with single giant liposomes embedded in agarose gel. Colloid Polym. Sci..

[47] Mabrouk E., Cuvelier D., Pontani L.-L., Xu B., Lévy D., Keller P., Brochard-Wyart F., Nassoy P., Li M.-H. (2009). Formation and material properties of giant liquid crystal polymersomes. Soft Matter.

[48] Mabrouk E., Cuvelier D., Brochard-Wyart F., Nassoy P., Li M.-H. (2009). Bursting of sensitive polymersomes induced by curling. Proc. Natl. Acad. Sci. USA.

[49] Marguet M., Sandre O., Lecommandoux S. (2012). Polymersomes in “Gelly” Polymersomes: Toward Structural Cell Mimicry. Langmuir.

[50] Marguet M., Edembe L., Lecommandoux S. (2012). Polymersomes in Polymersomes: Multiple Loading and Permeability Control. Angew. Chem..

[51] Peyret A., Ibarboure E., Pippa N., Lecommandoux S. (2017). Liposomes in Polymersomes: Multicompartment System with Temperature-Triggered Release. Langmuir.

[52] Chiba M., Miyazaki M., Ishiwata S. (2014). Quantitative analysis of the lamellarity of giant liposomes prepared by the inverted emulsion method. Biophys. J..

[53] Yamazaki M. (2008). Advances in Planar Lipid Bilayers and Liposomes.

[54] Elani Y., Law R., Ces O. (2014). Vesicle-based artificial cells as chemical microreactors with spatially segregated reaction pathways. Nat. Commun..

[55] Sugiyama H., Osaki T., Takeuchi S., Toyota T. (2020). Perfusion Chamber for Observing a Liposome-Based Cell Model Prepared by a Water-in-Oil Emulsion Transfer Method. ACS Omega.

[56] Kamiya K., Kawano R., Osaki T., Akiyoshi K., Takeuchi S. (2016). Cell-sized asymmetric lipid vesicles facilitate the investigation of asymmetric membranes. Nat. Chem..

[57] Lei L., Ma Y., Kodali D.R., Liang J., Davis H.T. (2003). Ternary phase diagram of soybean phosphatidylcholine-water-soybean oil and its application to the water degumming process. J. Am. Oil Chem. Soc..

[58] Shimane Y., Kuruma Y. (2022). Rapid and Facile Preparation of Giant Vesicles by the Droplet Transfer Method for Artificial Cell Construction. Front. Bioeng. Biotechnol..

[59] Tsumoto K., Hayashi Y., Tabata J., Tomita M. (2018). A reverse-phase method revisited: Rapid high-yield preparation of giant unilamellar vesicles (GUVs) using emulsification followed by centrifugation. Colloids Surf. A Physicochem. Eng. Asp..

[60] Lach S., Yoonm S.M., Grzybowski B.A. (2016). Tactic, reactive, and functional droplets outside of equilibrium. Chem. Soc. Rev..

[61] Sato Y., Takinoue M. (2019). Creation of artificial cell-like structures promoted by microfluidics technologies. Micromachines.

[62] Robinson T. (2019). Microfluidic handling and analysis of giant vesicles for use as artificial cells: A review. Adv. Biosyst..

[63] Lyu Y., Peng R., Liu H., Kuai H., Mo L., Han D., Li J., Tan W. (2020). Protocells programmed through artificial reaction networks. Chem. Sci..

[64] Olivi L., Berger M., Creyghton R.N.P., De Franceschi N., Dekker C., Mulder B.M., Claassens N.J., ten Wolde P.R., van der Oost J. (2021). Towards a synthetic cell cycle. Nat. Commun..

[65] Wang X., Tian L., Du H., Li M., Mu W., Drinkwater B.W., Han X., Mann S. (2019). Chemical communication in spatially organized protocell colonies and protocell/living cell micro-arrays. Chem. Sci..

[66] Li Q., Li S., Zhang X., Xu W., Han X. (2020). Programmed magnetic manipulation of vesicles into spatially coded prototissue architectures arrays. Nat. Commun..

[67] Villar G., Graham A.D., Bayley H. (2013). A tissue-like printed material. Science.

[68] Mukwaya V., Mann S., Dou H. (2021). Chemical communication at the synthetic cell/living cell interface. Commun. Chem..

[69] Murata S., Toyota T., Nomura S.M., Nakakuki T., Kuzuya A. (2022). Molecular Cybernetics: Challenges toward Cellular Chemical Artificial Intelligence. Adv. Funct. Mater..

[70] Takiguchi K., Yamada A., Negishi M., Tanaka-Takiguchi Y., Yoshikawa K. (2008). Entrapping desired amounts of actin filaments and molecular motor proteins in giant liposomes. Langmuir.

[71] Tanaka S., Takiguchi K., Hayashi M. (2018). Repetitive stretching of giant liposomes utilizing the nematic alignment of confined actin. Commun. Phys..

[72] Nishimura K., Matsuura T., Nishimura K., Sunami T., Suzuki H., Yomo T. (2012). Cell-free protein synthesis inside giant unilamellar vesicles analyzed by flow cytometry. Langmuir.

[73] Noireaux V., Libchaber A. (2004). A vesicle bioreactor as a step toward an artificial cell assembly. Proc. Natl. Acad. Sci. USA.

[74] Berhanu S., Ueda T., Kuruma Y. (2019). Artificial photosynthetic cell producing energy for protein synthesis. Nat. Commun..

[75] Sato Y., Komiya K., Kawamata I., Murata S., Nomura S.I.M. (2019). Isothermal amplification of specific DNA molecules inside giant unilamellar vesicles. Chem. Commun..

[76] Sato Y., Hiratsuka Y., Kawamata I., Murata S., Nomura S.I.M. (2017). Micrometer-sized molecular robot changes its shape in response to signal molecules. Sci. Robot..

[77] Shiomi H., Tsuda S., Suzuki H., Yomo T. (2014). Liposome-based liquid handling platform featuring addition, mixing, and aliquoting of femtoliter volumes. PLoS ONE.

[78] Terasawa H., Nishimura K., Suzuki H., Yomo T. (2012). Coupling of the fusion and budding of giant phospholipid vesicles containing macromolecules. Proc. Natl. Acad. Sci. USA.

[79] Schmid Y.R.F., Scheller L., Buchmann S., Dittrich P.S. (2020). Calcium-Mediated Liposome Fusion to Engineer Giant Lipid Vesicles with Cytosolic Proteins and Reconstituted Mammalian Proteins. Adv. Biosyst..

[80] Saito A.C., Ogura T., Fujiwara K., Murata S., Nomura S.M. (2014). Introducing micrometer-sized artificial objects into live cells: A method for cell–giant unilamellar vesicle electrofusion. PLoS ONE.

[81] Natsume Y., Noguchi E., Kurihara K. (2019). Spontaneous localization of particles in giant vesicles owing to depletion force. J. Phys. Soc. Jpn..

[82] Natsume Y., Toyota T. (2016). Asymmetrical polyhedral configuration of giant vesicles induced by orderly array of encapsulated colloidal particles. PLoS ONE.

[83] Suzuki K., Machida K., Yamaguchi K., Sugawara T. (2018). Photo-triggered recognition between host and guest compounds in a giant vesicle encapsulating photo-pierceable vesicles. Chem. Phys. Lipids.

[84] Bolognesi G., Friddin M.S., Salehi-Reyhani A., Barlow N.E., Brooks N.J., Ces O., Elani Y. (2018). Sculpting and fusing biomimetic vesicle networks using optical tweezers. Nat. Commun..

[85] Buddingh’ B.C., Elzinga J., van Hest J.C. (2020). Intercellular communication between artificial cells by allosteric amplification of a molecular signal. Nat. Commun..

[86] Hayashi H., Toyota T., Goto S., Ooishi A., Gao T., Ee L.B., Hatayama H., Nomoto T., Fujinami M., Matsubara H. (2015). Development of a non-blurring, dual-imaging tissue marker for gastrointestinal tumor localization. Surg. Endosc..

[87] Hatayama H., Toyota T., Hayashi H., Nomoto T., Fujinami M. (2014). Application of a novel near infrared-fluorescence giant vesicle-and polymerasome-based tissue marker for endoscopic and laparoscopic navigation. Anal. Sci..

[88] Su J., Kitaguchi T., Ohmuro-Matsuyama Y., Seah T., Ghadessy J.F., Hoon S., Ueda H. (2019). Transmembrane signaling on a protocell: Creation of receptor-enzyme chimeras for immunodetection of specific antibodies and antigens. Sci. Rep..

[89] Xu C., Lu P., Gamal El-Din T.M., Pei X.Y., Johnson M.C., Uyeda A., Bick M.J., Xu Q., Jiang D., Bai H. (2020). Computational design of transmembrane pores. Nature.

[90] Krinsky N., Kaduri M., Zinger A., Shainsky-Roitman J., Goldfeder M., Benhar I., Hershkovitz D., Schroeder A. (2018). Synthetic Cells Synthesize Therapeutic Proteins inside Tumors. Adv. Healthc. Mater..

[91] Toparlak Ö.D., Zasso J., Bridi S., Serra M.D., Macchi P., Conti L., Baudet M., Mansy S. (2020). Artificial cells drive neural differentiation. Sci. Adv..

